# The Effect of Exercise Intensity on Endothelial Function in Physically Inactive Lean and Obese Adults

**DOI:** 10.1371/journal.pone.0085450

**Published:** 2014-01-20

**Authors:** Rachel Hallmark, James T. Patrie, Zhenqi Liu, Glenn A. Gaesser, Eugene J. Barrett, Arthur Weltman

**Affiliations:** 1 Department of Human Services, University of Virginia, Charlottesville, Virginia, United States of America; 2 Department of Public Health Sciences, University of Virginia, Charlottesville, Virginia, United States of America; 3 Department of Medicine, University of Virginia, Charlottesville, Virginia, United States of America; 4 General Clinical Research Center, University of Virginia, Charlottesville, Virginia, United States of America; Medical University Innsbruck, Austria

## Abstract

**Purpose:**

To examine the effects of exercise intensity on acute changes in endothelial function in lean and obese adults.

**Methods:**

Sixteen lean (BMI <25, age 23±3 yr) and 10 obese (BMI >30, age 26±6 yr) physically inactive adults were studied during 3 randomized admissions [control (C, no exercise), moderate-intensity exercise (M, @ lactate threshold (LT)) and high-intensity exercise (H, midway between LT and VO_2_peak) (30 min)]. Endothelial function was assessed by flow-mediated dilation (FMD) at baseline and 1, 2, and 4 h post-exercise.

**Results:**

RM ANCOVA revealed significant main effects for group, time, and group x condition interaction (p<0.05). A diurnal increase in FMD was observed in lean but not obese subjects. Lean subjects exhibited greater increases in FMD than obese subjects (p = 0.0005). In the obese group a trend was observed for increases in FMD at 2- and 4-hr after M (p = 0.08). For lean subjects, FMD was significantly elevated at all time points after H. The increase in FMD after H in lean subjects (3.2±0.5%) was greater than after both C (1.7±0.4%, p = 0.015) and M (1.4±0.4%, p = 0.002). FMD responses of lean and obese subjects significantly differed after C and H, but not after M.

**Conclusion:**

In lean young adults, high-intensity exercise acutely enhances endothelial function, while moderate-intensity exercise has no significant effect above that seen in the absence of exercise. The FMD response of obese adults is blunted compared to lean adults. Diurnal variation should be considered when examining the effects of acute exercise on FMD.

## Introduction

Vascular endothelial dysfunction is a powerful non-traditional risk factor for cardiovascular disease (CVD) that is present prior to the development of atherosclerotic plaques [1]. Endothelial dysfunction is considered an important early indicator of atherogenic potential, as it is observed in the absence of frank atherosclerosis [2]. Brachial artery flow-mediated dilation (FMD) is a noninvasive procedure to assess endothelial function [1], and a meta-analysis demonstrated that impairment of brachial artery FMD is significantly associated with future cardiac morbidity and mortality [3]. Identification of this pre-atherosclerotic state could represent an important opportunity for disease-modifying interventions.

Exercise is one such intervention, and exercise training has been demonstrated to improve endothelial function in both healthy and unhealthy populations [4]. Additionally, single bouts of aerobic exercise affect endothelial function, although the results have been inconsistent. An acute bout of exercise has been reported to impair [5]–[9], enhance [10]–[14], or have no effect [6], [8], [11] on postexercise endothelial function. Aerobic exercise following caloric challenges also has been reported to mitigate the impaired FMD following an oral glucose tolerance test [15] and ingestion of a high-fat meal [13].

Reasons for the inconsistent findings with acute exercise may be due in part to the intensity and duration of the exercise stimulus, the mode of exercise, the population studied, the time of day of the exercise, and timing of the postexercise evaluation. Most studies have evaluated the acute effects of exercise by assessing endothelial function within the first hour after exercise [5]–[9], [11], [14], [15]. However, it is possible that the beneficial effect of a single bout of exercise on endothelial function may not be apparent for several hours [13]. Furthermore, many of these studies did not include a no-exercise control condition when examining the effect of the acute exercise intervention. Diurnal variation in endothelial function is evident in some populations [16], [17], and the absence of a no-exercise control condition may complicate interpretation of postexercise assessment of endothelial function.

There is currently no consensus in the literature regarding the impact of exercise intensity on endothelial function during the acute postexercise period. High-intensity interval [8], continuous [18], maximal [9] and prolonged (e.g., marathon) [5] exercise may impair endothelial function immediately postexercise and within the first hour postexercise, although health status may play a role [9]. Three studies have compared different exercise intensities with regard to postexercise endothelial function [10], [11], [18]. Currie et al. [10] reported that in subjects with coronary artery disease, both moderate-intensity endurance exercise and high-intensity interval exercise improved endothelial function 1 h after exercise. Harris et al. [11] found that regardless of exercise intensity, endothelial function was augmented 1 h after exercise in physically active overweight men, but in contrast was impaired in physically inactive overweight men. More recently, Birk et al. [18] reported that, in healthy normal weight adults, brachial artery FMD was impaired in an intensity-dependent manner immediately following 30 min of exercise at 50, 70, and 85% of HRmax. The effect of weight status on endothelial function responses to exercise is further complicated by the possibility that endothelial function in obese men may be enhanced with repeated assessments [14]. To our knowledge, no study to date has directly compared the effects of exercise intensity on endothelial function between subjects of different body composition, and in particular no study has controlled for the effects of diurnal variation on the endothelial response to exercise between lean and obese individuals.

In the present study we examined the effect of exercise intensity on acute changes in FMD in physically inactive lean and obese subjects. The changes following exercise were compared to the values observed during a no-exercise control day. Because shear stress on the vascular wall has been reported to mediate the endothelial adaptations to exercise training [19], and shear rate in the brachial artery increases with exercise intensity [20], we hypothesized that higher exercise intensity would be associated with a greater improvement in postexercise endothelial function. Also, because endothelial function is reduced in obesity [21]–[23], we hypothesized that the FMD response to acute exercise would be attenuated in physically inactive obese subjects.

## Methods

### Subjects

Healthy, but physically inactive non-smoking men and women, aged 18–35 years were recruited for this study. Subjects in the lean group were required to have a body mass index (BMI) <25 kg/m^2^, and a waist circumference of <80 cm for women or <94 cm for men. Obese subjects were defined as having a BMI >30 kg/m^2^, and a waist circumference of >80 cm for women or >94 cm for men was required to confirm abdominal adiposity. Subjects were excluded for current or recent performance of regular self reported endurance exercise training, defined as greater than 30 min of aerobic exercise on more than 2 days per week. Subjects were asked to maintain their normal diet and activity levels through the duration of the study. Women could not be pregnant or breastfeeding, and were required to have a regular menstrual cycle.

The present study was approved by the University of Virginia General Clinical Research Center Advisory Committee and the Human Investigation Committee (Institutional Review Board). All subjects provided written informed consent as approved by the Institutional Review Board, completed a medical history form, and underwent a physical exam performed by a study physician. Exclusion criteria included hypertension, diabetes, metabolic syndrome, severe pulmonary disease, coronary artery disease, or other cardiovascular disease. Subjects could not be taking medications used in the treatment of cardiovascular disease (including, but not limited to Angiotensin Converting Enzyme [ACE] inhibitors, Angiotensin Receptor Blockers [ARBs], HMG-coA reductase inhibitors [statins], phosphodiesterase inhibitors, beta-blockers, or alpha-blockers), or allopurinol.

Prior to the inpatient admissions, subjects completed outpatient testing of body composition and a lactate threshold/peak oxygen uptake (VO_2_peak) test on a cycle ergometer for determination of individual work rates during the exercise admissions. Subjects were also familiarized with the exercise protocol for subsequent visits.

### Anthropometrics and Body Composition

Weight was assessed on a calibrated electronic scale accurate to 0.01 kg (Life Measurement Instruments, Concord, CA). Height was measured using a calibrated stadiometer. Body density was measured by air displacement plethysmography (BodPod Life Measurement Instruments, Concord, CA), corrected for thoracic gas volume [24]. A 2-compartment model was used to assess body composition, and the *Siri equation* [25] was used to determine percent body fat from body density measurements. Waist circumference was measured in triplicate at the umbilicus using cloth tape with a spring-loaded handle.

### Lactate Threshold and Peak Oxygen Uptake (VO_2_peak)

Subjects began cycling at an initial power output of 20 W, and the power output was increased by 15 W every 3 min (the duration of each stage), until volitional exhaustion. Metabolic measures were determined using open-circuit spirometry (SensorMedics Model 229 metabolic measurement cart, Yorba Linda, CA), and heart rate was assessed continuously by 12-lead EKG (Marquette Max-1, Marquette, WI). At baseline (0 min) and during the last minute of each stage, blood was sampled from an IV catheter in the subject’s hand or forearm for the measurement of blood lactate concentration (YSI Instruments 2700, Yellow Springs, OH). The lactate threshold (LT) was determined from the blood lactate-power output relationship, and was defined as the highest power output attained prior to the curvilinear increase in blood lactate concentration above baseline. A lactate elevation of at least 0.2 mM (the error associated with the lactate analyzer) was required for LT determination [26]. Individual plots of VO_2_ vs. power output were used in the determination of the VO_2_ associated with the LT. Moderate-intensity exercise was chosen as the power output associated with the LT and high-intensity exercise was chosen as the power output midway between the LT and peak power output. Blood lactate was measured every 10 min during the 30 min constant-power exercise bouts (moderate- and high- intensity admissions), to ensure the appropriate intensity of exercise.

### Study Design

This study was performed in a randomized crossover design. Each subject was admitted to the General Clinical Research Center during the evening prior to study, on three separate occasions (control, moderate-intensity exercise, and high-intensity exercise). Study days were scheduled at least 48 h apart, to avoid potential carryover effects from prior exercise testing. Women were studied during the follicular phase (days 2–8) of the menstrual cycle to control for potential effects of hormonal variation on vascular responsiveness [27].

Subjects were fasted for 12 h prior to the start of each testing day, and no food or drink (except water) was permitted until test completion. No alcohol or caffeine was permitted for 24 h prior to each test day. Subjects were asked to refrain from strenuous physical activity for 48 h prior to each test day, to avoid potential carry-over effects from prior activity. Subjects were also instructed not to take anti-histamines for 5 days prior to each admission, to avoid actions of these agents on the vasculature [28]. Oral vitamin C supplementation was restricted for 2–7 days prior to study, depending on the dosage typically consumed.

In the morning of each test day, an intravenous (IV) catheter was placed in the subject’s dominant hand or arm. The first flow-mediated dilation (FMD) measurement was performed between 0700–0800. Following the baseline measurement, subjects completed 30 min of cycle exercise at either a moderate- or high-intensity, or rested upright in a chair for 30 min (control). Following the exercise or control period subjects returned to their room and remained in bed in supine rest for the remainder of the study. FMD was repeated 1, 2, and 4 h postexercise. Blood pressure was measured at baseline and hourly post-exercise, with the subject lying supine with legs uncrossed, using a manual sphygmomanometer. The systolic (SBP) and diastolic blood pressure (DBP) values measured at the initial screening visit, at each time point on the control day, and at baseline on the moderate- and high-intensity days, were averaged to create a mean value for each subject.

### Flow-Mediated Dilation (FMD)

Flow-mediated dilation was performed to determine the nitric oxide (NO)-dependent vasodilatory capacity of the brachial artery. Images of the brachial artery were obtained using high-resolution 2D and Doppler ultrasound (HDI 5000, ATL, Philips Ultrasound, Andover, MA) with a linear-array transducer at a transmit frequency of 12 MHz. Imaging was performed following 20 min of supine rest on each subject’s non-dominant arm, immobilized in an extended position. Images of the brachial artery were taken in the longitudinal plane, proximal to the antecubital fold. The precise location of the ultrasound probe was individualized to optimize image clarity, and to avoid areas of arterial branching. The location of the probe was marked during the first measurement of each test day, and repeat measurements were performed in the same region. In addition, anatomical landmarks visible on ultrasound were used whenever possible to ensure proper location of the probe between trials. Heart rate triggers were used to capture standardized images at the onset of the R-wave (end diastole), to avoid the effects of vasomotion associated with the cardiac cycle. Following image optimization (and before cuff inflation), three images were captured for determination of average brachial artery diameter. A blood pressure cuff (placed 2 cm distal to the antecubital fold) was then inflated to >200 mmHg for 5 min. Upon cuff release, peak blood flow was determined within the first 20 s of reactive hyperemia. Digital images of the artery were then captured every 5 s from 30 s to 120 s to determine peak dilation.

Digital images were analyzed with edge-detection software (Brachial Analyzer, Medical Imaging Applications, Iowa City, IA) by a single investigator, blinded to both time and condition. Arterial diameters (mm) were calculated as the mean distance between the anterior and posterior wall at the intima-lumen interface. Percent FMD is defined as the change in vessel diameter from rest to peak dilation as a percentage of the baseline diameter. Peak shear rate was calculated as the peak hyperemic velocity (cm/s) divided by the baseline vessel diameter (cm), as suggested by Pyke and Tschakovsky [29]. The use of edge-detection software significantly minimizes investigator bias and has a mean reported intraobserver coefficient of variation of 6.7% for repeated measures of FMD, which is significantly lower than the traditional manual, on-line caliper method [30]. In our lab, the intraobserver variability is 2.93% [31].

### Statistical Analysis

Differences in the descriptive characteristics of the two groups were analyzed by a Student’s unpaired *t*-test. Percent FMD data were analyzed using a linear mixed-effects ANCOVA model. The response data was the change in %FMD at 1, 2 and 4-h post-intervention relative to the baseline FMD. Three potential sources of measurement variability were evaluated in the ANCOVA: 1) obesity status (abdominally obese or lean), 2) condition (control, moderate-intensity, or high-intensity exercise), and 3) measurement time (1, 2, or 4 h). All 2-way and 3-way interactions were also examined as potential sources of measurement variability. The baseline FMD measurements served as the ANCOVA covariate adjustment factor.

Fisher’s Restricted Least Significant Difference (RLSD) criterion was utilized to maintain an overall type I error rate of 0.05. Multiple degrees of freedom F-tests were used to evaluate the significance of 2- and 3-way interactions. Pairwise comparison testing was carried out by way of linear contrasts of the least-squares means. The decision rule for rejecting the null hypothesis was based on the two-sided P≤0.05 criteria. The Student’s t-distribution multiplier was used to determine the lower and the upper limits of each 95% confidence interval.

Measurements of the change in shear rate from baseline were analyzed on the natural logarithmic scale via a linear mixed-effects model. Measurements of the pre-FMD brachial artery diameter were also analyzed via a linear-mixed effects model. Three sources of variation in the response measurements were examined for the measurements of shear rate and pre-FMD diameter, corresponding to the analysis of changes in %FMD: 1) obesity status (abdominally obese or lean), 2) condition (control, moderate-intensity, or high-intensity exercise), and 3) measurement time (1, 2, or 4 h). Hypothesis testing was conducted via F-tests.

Statistical analysis was performed using the PROC MIXED procedure of SAS version 9.1.3 EAS (SAS, Institute Inc, Cary NC).

## Results

### Subject Characteristics

Descriptive characteristics of the two subject groups are summarized in Table 1. By design, significant differences were observed in all measures of body composition: obese subjects had significantly elevated BMI, % body fat, and waist circumference compared to lean subjects (p<0.001). Groups differed significantly in systolic (p<0.001) and diastolic (p = 0.01) blood pressure at rest, but both groups were normotensive. There was also a significant difference between the groups in VO_2_peak, normalized to either total body weight (mL/kg/min, p = 0.0002) or to fat-free mass (mL/kg FFM/min, p = 0.008). Among lean subjects, moderate- and high-intensity exercise occurred at 45.4±12.3 and 72.8±7.7% of VO_2_peak, respectively. For the obese subjects, these exercise intensities were associated with 50.9±11.0 and 75.4±11.6% of VO_2_peak, respectively.

**Table 1:** Subject Characteristics.

	Lean	Obese
Males/Females	8/8	4/6
Age (yr)	23±3	26±6
BMI (kg/m^2^)	22.6±2.1	36.4±7.0*
Waist Circumference (cm)	85.5±17.5	116.1±20.4*
% Body Fat	24.3±7.8	39.4±11.9*
Systolic BP (mm/Hg)	106±7	118±9*
Diastolic BP (mm/Hg)	66±5	75±11*
VO_2_peak (ml/kg/min)	29.8±6.3	19.2±4.9*
VO_2_peak (ml/kgFFM/min)	39.2±6.8	31.7±5.7*

Data presented are mean±SD,

p<0.05.

### Flow-Mediated Dilation (FMD)

**Figure 1:**
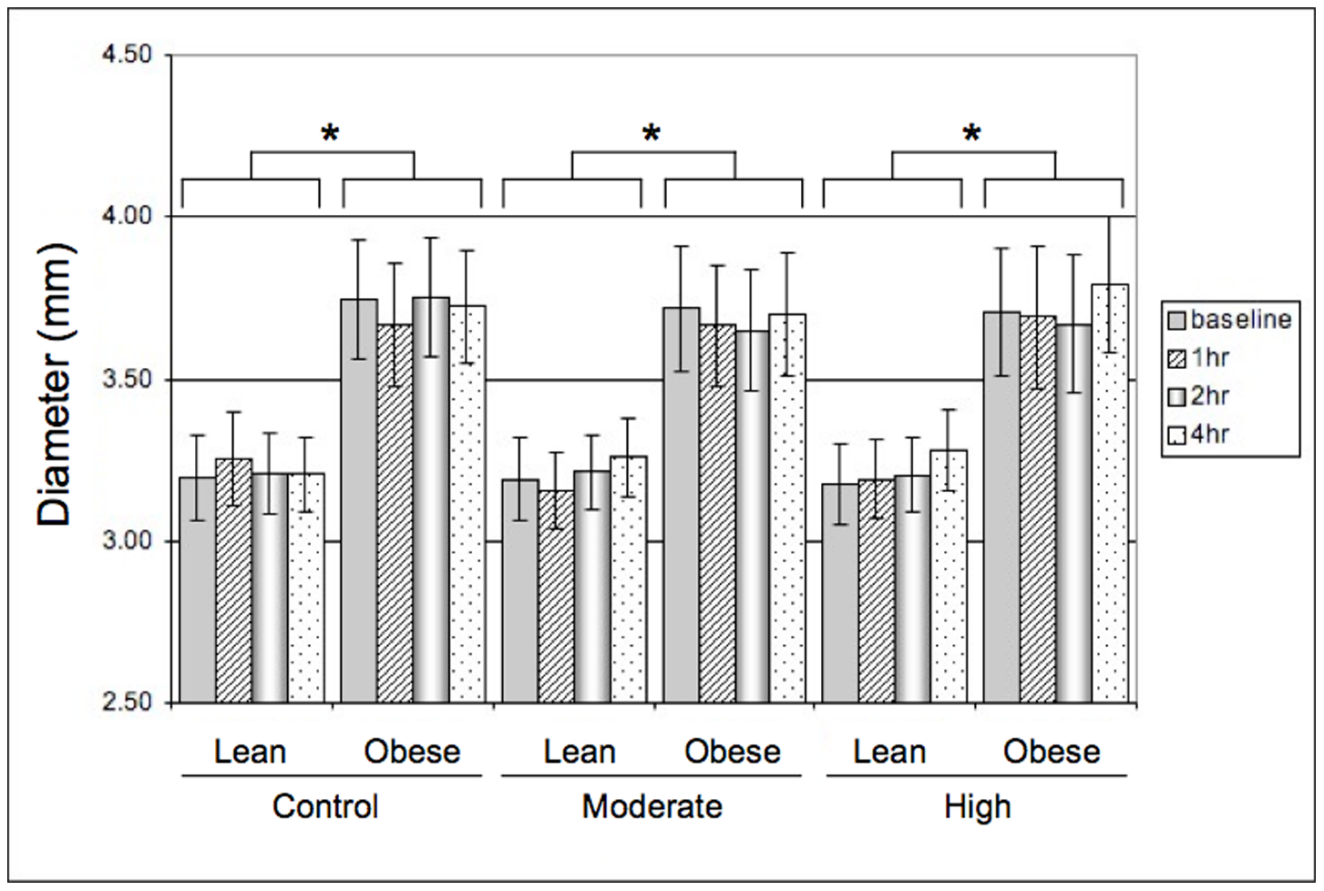
Baseline (pre-cuff inflation) brachial artery diameter (mean±SE; * P<0.05 lean vs obese).

The pre-FMD brachial artery diameter measurements are shown in Figure 1. For the 1 h time point during the control day, there were missing data for 3 male subjects (1 obese, 2 lean). These data points were omitted from the pre-FMD diameter analysis as well as the FMD analyses (below), with resulting sample sizes of n = 14 lean and n = 9 obese subjects for this time point only. The pre-FMD brachial artery diameter differed significantly between lean and obese adults for all conditions (p<0.05). However, there were no significant differences in pre-FMD brachial artery diameter within groups, either between conditions or over time.

**Figure 2:**
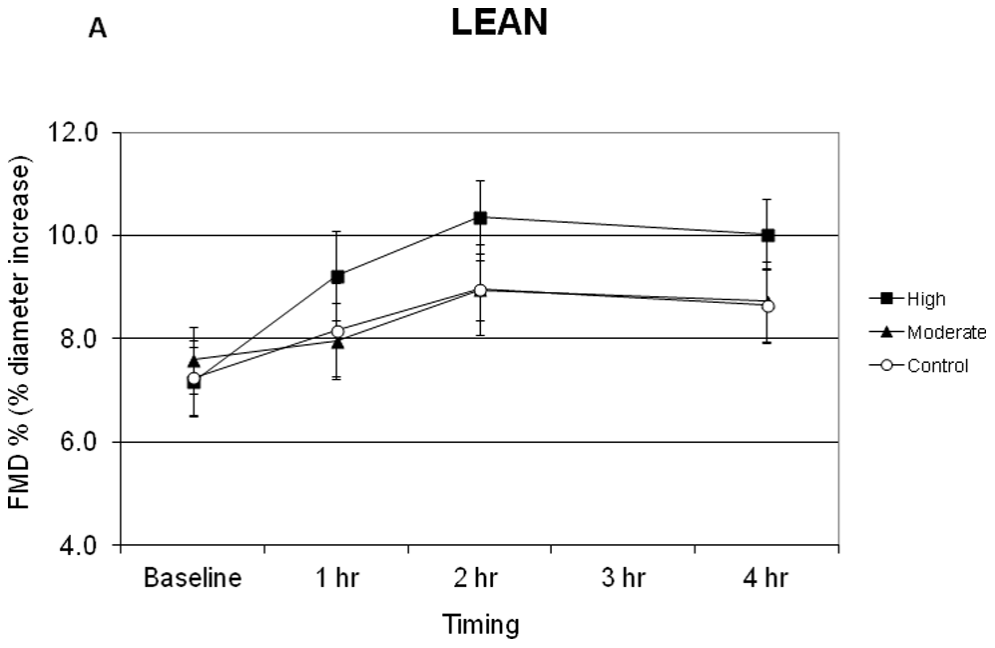
Flow mediated dilation (FMD) responses of lean subjects during the control moderate intensity and high intensity exercise conditions.

**Figure 3:**
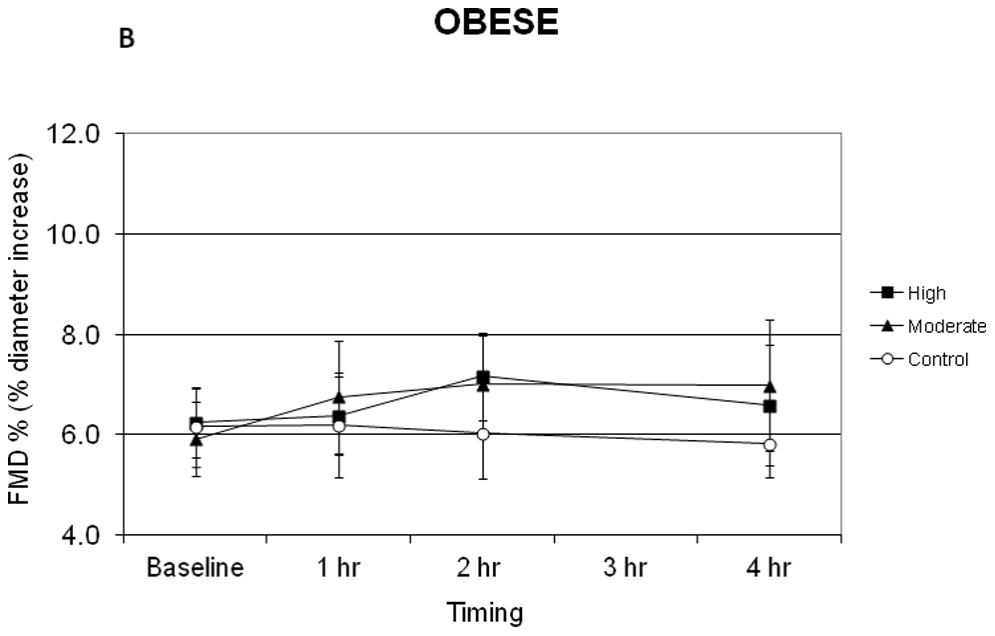
Flow mediated dilation (FMD) responses of obese subjects during the control moderate intensity and high intensity exercise conditions. Statistical analyses revealed the following: No significant differences between lean and obese subjects at baseline for any condition; FMD in lean subjects increased significantly compared to baseline at 2 h and 4 h in the absence of exercise (p<0.02) with no change observed in obese subjects; FMD change over time in lean subjects was greater than obese subjects during the no exercise control condition (p = 0.011); following high-intensity exercise, FMD in lean subjects was increased significantly compared to baseline at all time points (p<0.001) and these values were greater than those achieved on the control day (p<0.02) and greater than the peak values observed in obese subjects (p<0.0005).

Figures 2 and 3 display the mean FMD responses of lean and obese subjects during the control and exercise conditions. Although the average baseline FMD across conditions was 1.074% higher in lean compared to obese subjects these differences did not reach the level of statistical significance.

In the control condition, FMD in lean subjects was increased significantly compared to baseline at 2 h and 4 h in the absence of exercise (Figure 2, p<0.02), and the increase at 1 h approached statistical significance (p = 0.053). In contrast, the FMD of obese subjects (Figure 3) did not change significantly from baseline values across the control measurement period. Thus FMD values of the lean group were significantly greater than the obese group during the control condition (p = 0.011), with the lean subjects achieving a mean increase of 1.7±0.4% (relative increase of 26%) above baseline FMD during the control condition (Figure 2).

After moderate-intensity exercise, FMD in lean subjects was significantly increased above baseline values at 2 h and 4 h (p<0.02), but these values were not different from those observed on the control day in the absence of exercise (Figure 2). The FMD of obese subjects was not significantly increased above baseline for any of the time points following moderate-intensity exercise, but a trend was observed for an increase in FMD at 2 h and 4 h post-exercise (Figure 3, p = 0.076 and p = 0.082, respectively). No statistically significant differences were observed between the change in FMD values of lean and obese subjects after moderate-intensity exercise (Figures 2 and 3, maximum increase of 1.4% and 1.1% above baseline, respectively).

Following high-intensity exercise, FMD in lean subjects was increased significantly compared to baseline at all time points (Figure 2, p<0.001), and these values were also significantly greater than those achieved on the control day (p<0.02). FMD did not increase significantly over time following high-intensity exercise in the obese group (Figure 3, p = 0.82, 0.13, 0.56 for 1 h, 2 h, and 4 h, respectively). Thus the mean FMD increase of 3.2±0.5% (relative increase of 59%) above baseline after high-intensity exercise in the lean group was significantly greater than the peak increase above baseline of 0.9±0.4% observed in obese group (p<0.0005).

## Discussion

The primary findings of the present study are that exercise intensity is an important determinant of the acute FMD response in lean adults, and that lean, but not obese, adults demonstrate a diurnal variation in FMD (at least in morning). The latter finding indicates the importance of including a no-exercise control day for interpreting FMD responses during a post-exercise period lasting several hours. Our results also suggest that obese adults may have a blunted FMD response to moderate- and high-intensity exercise.

The effect of exercise intensity on postexercise FMD is unclear. Some studies have found that very high-intensity exercise acutely impairs endothelial function. As examples, brachial artery FMD was reduced 1 h after high-intensity interval exercise in endurance-trained men [8], immediately after a marathon race in non-elite male runners [5], in an intensity-dependent manner immediately after 30 min of exercise in healthy men [14], and after maximal treadmill exercise in patients with intermittent claudication [9]. In contrast, exercise for 45 min at 60% VO_2_peak increased FMD 1 h post-exercise in postmenopausal women [12] and 4 h postexercise in healthy men and women [13]. Furthermore, a recent study reported that both moderate-intensity continuous exercise and high-intensity interval exercise resulted in similar improvements in FMD 1 h post-exercise in subjects with coronary artery disease [10]. Finally, although Birk et al. [18] reported that FMD was impaired immediately post-exercise, their data also indicated that FMD returned to baseline within 1 h after exercise, independent of exercise intensity. Their observations, as well as the results of the present study, support the importance of standardizing the time between the cessation of exercise and post-exercise FMD measurement. Had we not included a no-exercise control condition and examined the effect of exercise on FMD with respect to the baseline FMD value only, our results for moderate-intensity exercise (∼45–50% VO_2_peak) in lean subjects would be consistent with previous findings [12], [13]. However, in lean subjects the change in FMD after moderate-intensity exercise (maximum increase = 1.4%) was essentially the same as that observed in the absence of an exercise stimulus (1.7% increase). Previous studies [12], [13] did not use a no-exercise control trial, and thus the magnitude of the increase in FMD attributed to acute exercise alone may be overstated. This would be especially true when FMD is measured 4 h after exercise [25], but less likely to undermine conclusions about FMD taken within 1 h after exercise [14].

In our study, high-intensity exercise elicited 73–75% VO_2_peak for both lean and obese subjects. The increase in FMD at 1, 2, and 4 h after high-intensity exercise was evident only in lean subjects. In contrast, Zhu et al. [14] recently reported that in obese young men, 45 min of exercise at 80% of age-predicted maximum HR (∼70–75% of VO_2_max) increased FMD 1 h and 2 h post-exercise. In their subjects, the increases in FMD 1 h and 2 h post-baseline were ∼1.5% FMD greater after exercise than during the control trial, but no statistical analysis was reported to reveal whether these were significantly different. One possible explanation for the different findings is that the exercise duration was 50% longer in the study of Zhu et al. [14]. We are not aware of any studies that have systematically evaluated the effects of exercise duration on FMD.

Previous work in humans and animals has suggested the existence of an intensity-dependent increase in endothelial function after exercise. Green et al. [32] demonstrated that systemic, NO-dependent exercise hyperemia depends upon combined increases in blood pressure and heart rate (HR), and is not achieved with increases in HR alone. Handgrip exercise elicits a smaller magnitude of brachial artery hyperemia than lower-body cycle exercise [33], and has failed to improve systemic endothelial function [34]. These observations suggest that a critical threshold of exercise intensity may exist for endothelial adaptations, which may be related to elevations in blood pressure and associated increases in shear stress. However, the specific exercise intensity threshold is currently unknown, and may depend on the baseline status of endothelial function for the population in question.

FMD did not increase significantly from baseline values in any of the three conditions in our obese subjects (Figure 3). Harris et al. [11] also reported that low-, moderate-, or high-intensity treadmill walking did not increase FMD in overweight men. A significant improvement in FMD was only observed after pooling the results of the 3 intensities, and this was only found in physically active overweight men [11]. In the present study we observed an increase in FMD at 2 h and 4 h after moderate-intensity exercise in obese subjects, which approached statistical significance. FMD responses of lean and obese adults to moderate-intensity exercise were not significantly different (Figures 2 and 3). However, due to the divergent patterns of FMD in lean and obese adults on the control day, it is essential to compare the post-exercise FMD to changes observed in the absence of exercise. In obese subjects, moderate-intensity exercise increased FMD by an average of 1.1% over baseline, compared to essentially no change during the control day (−0.1 to −0.3%). Following high-intensity exercise, FMD increased by 0.9% in obese subjects. Thus, it is possible that acute exercise may modestly enhance FMD in obese subjects independent of exercise intensity.

The cause for the divergence of lean and obese responses to acute high-intensity exercise is not known. The current study was not designed to investigate the mechanistic explanation for the observed differences, but several possibilities can be proposed. In both sedentary and endurance trained men and women, there is a histamine-mediated mechanism associated with post-exercise hyperemia [35]. Whether this response is blunted in obesity is unknown. It is known that both BMI and % body fat are inversely related to eNOS protein content and enzymatic activity in skeletal muscle [36]. This could result in a decreased capacity for NO production in obese subjects. In addition, the release of endothelin-1 (ET-1) from adipose tissue is reported to be 2.5-fold higher in obese compared to lean subjects at rest [37]. If this vasoconstrictive pathway is similarly enhanced in obese subjects after exercise, it might partially abrogate the vasodilation induced by NO production. Finally, previous work suggests that production of reactive oxygen species (ROS) in response to exercise may result in a reduction in NO availability [38] and that ROS production is greater in obese compared to lean adults [39]. Elevations in ROS have been suggested as a potential mechanism for the observed reduction in FMD immediately following high intensity exercise [18]. The differential production and possibly clearance of ROS during high-intensity exercise may have contributed to the observed responses in lean and obese adults by altering the bioavailability of circulating NO. These mechanistic possibilities regarding the obesity-dependent effects of exercise on FMD remain to be tested.

It is also important to note that the baseline diameter of arteries from lean and obese subjects were significantly different in the present study (Figure 1). Although smaller vessels dilate to a greater extent than larger vessels, when expressed as a percentage of baseline diameter, this effect of arterial diameter is not related to differences in arterial shear stress elicited during FMD [40]. In the current study, FMD was not related to either peak arterial shear rate or baseline arterial diameter. In addition, while significant differences existed between the lean and obese groups for baseline diameter, the within-group diameter values did not change significantly over time in either the presence or absence of exercise (Figure 1). The effect of obesity on endothelial function in general may relate to the larger arterial diameter in this population compared to lean subjects. Consistent with the larger arterial diameter in obese subjects, we found that baseline FMD was on average 1.074% lower in obese subjects. Although this difference did not reach the level of statistical significance, the findings are consistent with previous literature that indicates that FMD is impaired in obesity [21]–[23]. However, the different patterns of post-exercise FMD in lean and obese subjects cannot be attributed to diameter effects.

In the present study, FMD values were not normalized to the magnitude of shear stress or shear rate, as has been suggested by Pike et al. [29], [41]. The area under the curve (AUC) of the shear profile up to the point of peak dilation is suggested to be a predictor of FMD by some authors [41], but the basis of this relationship has been repeatedly called into question [40], [42]. The issue of normalizing FMD to shear is currently unresolved [42]. Our ultrasound system is not configured for simultaneous measurement of diameter and blood flow, so only the peak shear rate could be determined. It has been suggested that *peak* shear rate is not an important determinant of FMD responses [28] and in the current study peak shear rate was not different between lean and obese groups (data not shown), and this value was not associated with FMD.

Furthermore, it should also be noted that the value of FMD as a predictor of future CVD risk and mortality has been validated only in its original (i.e. non-normalized) formulation [2].

A diurnal variation in FMD has been reported in healthy adults [16], [17], with FMD typically being lowest in early morning hours and then increasing throughout the morning and afternoon [16]. The current results confirm a diurnal variation in FMD in lean adults, but such a pattern was not observed in the obese subjects. Our findings are in contrast to those of Zhu et al. [14], who reported that FMD increased from 5.9% to 8.4% during a 2-h period in the morning in obese young men but did not in lean young men. Zhu et al. (14) did not attribute the increase in FMD to a diurnal cause but rather to the effects of repeated FMD procedures. Our data provide no support for that conclusion (Figures 2 and 3). Furthermore, repeated FMD procedures do not appear to influence the FMD response [43].

The lack of difference between the FMD profile of lean and obese adults following moderate-intensity exercise must also be interpreted in the context of significant differences in FMD observed during the no-exercise control condition. In obese subjects, moderate-intensity exercise trended toward a modest increase in FMD (1.5% difference between moderate-intensity and control), while no change was observed in lean subjects (−0.3% difference). It is inappropriate to assume that the pattern of changes in FMD in the absence of exercise or another acute stimulus will be similar when comparing groups of subjects.

Not including a no-exercise control trial is most likely to affect interpretation of the effect of acute exercise on FMD when the post-exercise measurements are taken 2–4 h after exercise (Figures 2 and 3). Nonetheless, because FMD increased by ∼1% per h between approximately 8 and 10 AM in the absence of exercise (Figure 2), interpretation of small changes in FMD even in the first 1–2 h post-exercise may be compromised without inclusion of a no-exercise control trial. This might be most important when studying young healthy subjects, who are most likely to display diurnal variation in FMD [16], [17]. Previous studies assessing FMD up to 4 h post-exercise [13], [15] did not include a control trial.

Additional complexity can be encountered in non-interventional, cross-sectional analyses of FMD if this diurnal variation is not considered. For example, previous work has suggested that FMD is significantly correlated with BMI [44] and indices of abdominal adiposity, such as waist-to-hip ratio [22] or visceral fat content [21], [33]. In the present study the correlations between waist circumference and FMD measured at baseline and 1, 2, and 4 h after moderate and high intensity exercise was <r = 0.35. Average baseline FMD values for lean subjects were 1.074% higher than obese subjects and at all other times on the control day the FMD of lean subjects increased over baseline and were significantly greater than that of obese subjects. These results suggest that the timing of an FMD measurement may significantly impact the observed relationship between obesity status and endothelial function. Future studies should take into account potential differences in circadian variation when comparing endothelial function between groups.

### Conclusions

This study is the first to our knowledge to directly compare the responses of lean and obese adults to acute bouts of exercise of differing intensity, corrected for changes in non-exercise FMD over time. Lean and obese adults of low fitness exhibit different responses of endothelial function to an acute bout of moderate- or high-intensity exercise. Significant improvements in conduit artery endothelial function are observed following high- but not moderate-intensity exercise in lean subjects. In contrast, the FMD response of obese adults appears to be similar following moderate- and high-intensity exercise, and is blunted compared to lean adults. However, in light of the lack of change in FMD in obese subjects in the absence of exercise, the modest effects of exercise on vascular endothelial function in this population may be clinically relevant.
